# Effects of Yu-Ping-Feng polysaccharides on animal growth performance and immune function: a review

**DOI:** 10.3389/fvets.2023.1260208

**Published:** 2023-09-20

**Authors:** Huangbin Chu, Yi Zong, Hong Yang, Siyu Chen, Zheng Ma, Hua Li

**Affiliations:** Guangdong Provincial Key Laboratory of Animal Molecular Design and Precise Breeding, School of Life Science and Engineering, Foshan University, Foshan, Guangdong, China

**Keywords:** Yu-Ping-Feng polysaccharides, function, animal growth performance, animal immunity, animal health farming

## Abstract

*Yu-Ping-Feng polysaccharides (YPF-P)* is the primary component of *Yu-Ping-Feng San (YPF-S)* medicine prescription, which consists of three polysaccharides from *Astragalus Membranaceus polysaccharide* (*AM-P*), *Atractylodes Macrocephala polysaccharide* (*AM-P*), and *Saposhnikovia Divaricata polysaccharide* (*SD-P*). The use of Yu-Ping-Feng polysaccharides dates back to the Yuan Dynasty when Yilin Wei first utilized it. The remedy is included in “*Effective Formulae Handed Down for Generations*” and “*The Pharmacopoeia of the People’s Republic of China*.” Yu-Ping-Feng polysaccharides is known to promote growth and enhance the body’s immune function in animals. As such, it has promising application prospects in animal husbandry. This review mainly introduces the main components and characteristics of Yu-Ping-Feng polysaccharides, its effects on animal production, and its impact on animal immune function. Additionally, this paper offers a preliminary discussion on the development and utilization of Yu-Ping-Feng polysaccharides, laying the foundation for further research and application. This review may also provide insight and reference for the development of the farming industry, reducing production costs and improving productivity.

## 1. Introduction

In recent years, chemically synthesized antibiotics have been widely used in the field of economic animal husbandry for animal health improvement. However, these chemically synthesized formulations could lead to permanent residues within animals, posing potential health threats to both animals and humans.

Chinese herbal compound polysaccharides have demonstrated significant effects in immune regulation, antiviral activity, antioxidation, and blood glucose reduction, among other aspects. Moreover, due to the lack of toxic side effects on tissues and cells, these advantages contribute to a broad potential application of polysaccharides in antibiotic-free animal farming.

Research has confirmed that the Chinese herbal compound polysaccharide - specifically Yu-Ping-Feng polysaccharide - can enhance animal growth performance and immunity. It holds the potential to replace antibiotics and become a viable non-antibiotic feed additive. This article provides a comprehensive review of the composition, characteristics, and functions of Yu-Ping-Feng polysaccharides in animal production, offering insights for further theoretical research and practical applications in animal farming (1).

## 2. Composition and characteristics of Yu-Ping-Feng polysaccharides

Yu-Ping-Feng San is a traditional Chinese herbal compound preparation, composed of *Astragalus Membranaceus* (Huang qi), *Atractylodes Macrocephala* (Bai zhu), and *Saposhnikovia Divaricata* (Fang feng) in a ratio of 3:1:1 by weight. Yu-Ping-Feng San is a classical Chinese medicine formula that has a broad range of clinical applications and is effective in treating various diseases, including those related to immunity, inflammation, tumors, viral infections, and lungs (2–7).

**Figure 1:**
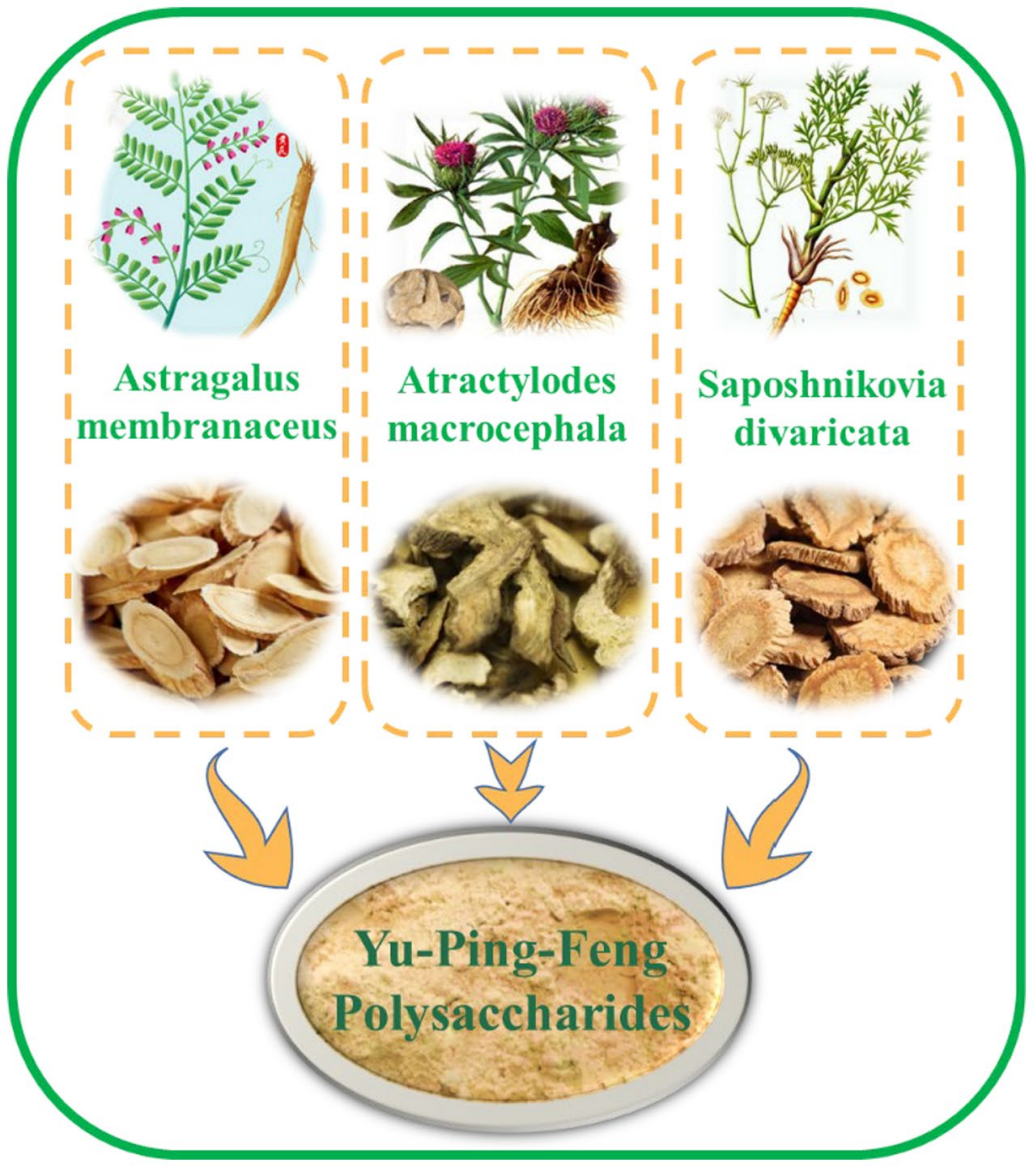
Yu-Ping-Feng polysaccharides is extracted from three traditional Chinese medicines.

Yu-Ping-Feng polysaccharides are primarily extracted from Yu-Ping-Feng dispersible granules using the “water decoction and alcohol precipitation method (8, 9) “They appear as a pale yellow to light brown powder, characterized by strong hygroscopicity and a tendency to clump. Yu-Ping-Feng polysaccharides are the main active components responsible for the therapeutic effects of Yu-Ping-Feng San (10–12). Yu-Ping-Feng polysaccharides mainly consist of three polysaccharide components: YPF-P1, YPF-P2, and YPF-P3. Among these, YPF-P1 is the most abundant, accounting for 76.1% of the total. YPF-P2 is present in a lower amount, constituting only 11.7%, while YPF-P3 comprises just 7.4% (11). As shown in Figure 1, Yu-Ping-Feng polysaccharides is extracted from a combination of three traditional Chinese medicinal ingredients.

Modern pharmacological research has revealed that the primary effects of Yu-Ping-Feng polysaccharides are to enhance overall human health. Yu-Ping-Feng polysaccharides can improve both specific and non-specific immunity, including erythrocyte immunity, and stimulate the proliferation of T lymphocytes. Furthermore, they exhibit antibacterial, antiviral, and antioxidative stress properties (13). Besides, Yu-Ping-Feng prescription has been confirmed to have certain therapeutic effects on respiratory system diseases such as respiratory tract infections and asthma, immunodeficiency diseases, as well as allergic diseases (14–19). To date, only a handful of mild to moderate adverse reactions have been reported due to the use of the Yu-Ping-Feng prescription. These reactions include thirst, dry tongue, acne, abdominal distension, gingivitis, toothache, water-sodium retention, and gastrointestinal bleeding, among others (20). The various benefits of the immunomodulatory effects exhibited by Yu-Ping-Feng polysaccharides are also applicable to animals. Relevant studies have been carried out on animals such as mice, piglets, chicks, and grass carp (1, 21–23).

## 3. The role of Yu-Ping-Feng polysaccharides in animal production

### 3.1. Effect of Yu-Ping-Feng polysaccharides on pig production performance

Yu-Ping-Feng polysaccharides can be used as a feed additive with feed. It has been found that the addition of Yu-Ping-Feng polysaccharides to sow diets could effectively alleviate the stress of gestating sows, improve their production and physiological functions, and significantly increase the farrowing survival rate, 7-day weaning rate, litter size, healthy litter size, litter weight of newborn piglets, and average weaning weight of sows (*p < 0.05*) (24). Yu-Ping-Feng polysaccharides also plays an important role in the production of growing fattening pigs, and it can effectively regulate the intestinal microbial community structure of pigs and promote their growth (25). And related studies showed that the addition of 0.8% Yu-Ping-Feng polysaccharides had a significant weight gain effect on growing pigs (*p < 0.05*) (26, 27). In a similar experiment, also with the addition of 0.8% Yu-Ping-Feng polysaccharide, the survival rate of the test group could be increased by 5.1 percentage points compared to the control group (28). In addition, the final body weight and daily weight gain of pigs were improved by adding Yu-Ping-Feng polysaccharides 100 mg/kg, 200 mg/kg, 400 mg/kg, and 600 mg/kg to Duroc× Landrace× Yorkshire growing-finishing pigs’ diets after 21 days of feeding, and the feed-to-weight ratio decreased with the increase of Yu-Ping-Feng polysaccharides addition (21). Other studies showed that probiotics and Yu-Ping-Feng polysaccharides were used separately and both improved the daily weight gain rate of ternary piglets (*p < 0.05*), however, the combination of Yu-Ping-Feng polysaccharides and probiotics was more beneficial for piglets (29). In conclusion, the addition of Yu-Ping-Feng polysaccharides to the diet can improve the productivity of pigs and has promising applications.

### 3.2. Effect of Yu-Ping-Feng polysaccharides on the production performance of poultry

The digestion and absorption of nutrients in chickens are mostly concentrated in the posterior end of the digestive tube (i.e., the small intestine). A related study demonstrated the effects of five Chinese herbals on chick growth and development and found that one of them, the Yu-Ping-Feng polysaccharides, promoted chick growth and development (30). Related research has shown that Yu-Ping-Feng polysaccharides is capable of effectively improving the structure of the intestinal flora in poultry, regulating the expression of genes (*SGLT1, GLUT2, GLUT5*) in chicken intestinal mucosal epithelial cells, and promoting faster nutrient absorption. As a result, feed utilization is enhanced and the growth characteristics of poultry are altered (25, 31). However, its digestive and absorptive capacity is affected during chick vaccination; therefore, some studies have attempted to add Yu-Ping-Feng polysaccharides to the diets of chicks during vaccination and found that it significantly reduced the meat-to-feed ratio of chicks (32). In addition, Yu-Ping-Feng polysaccharides has also been shown to have significant advantages in increasing body weight, improving the survival rate, and increasing feed intake in broilers (33–35). Interestingly, another study found that both Yu-Ping-Feng polysaccharides and antimicrobial peptides were able to improve feed conversion in broilers; However, the combination use of Yu-Ping-Feng polysaccharides and antimicrobial peptides can lead to reduced feed intake and survival rate in broilers (36). Subsequently, a search was made for substances that could be used in combination with Yu-Ping-Feng polysaccharides; it was found that the licorice preparation could improve the efficacy of Yu-Ping-Feng polysaccharides, and the simultaneous use of both preparations could significantly improve the growth performance of broiler chickens with a synergistic promotion effect (37). In addition, another study discussed the use of a combination of Yu-Ping-Feng polysaccharides and compound acidifier and discovered that this combination significantly improved the growth performance of white feather broiler chickens. The combination achieved this effect by regulating the intestinal pH and increasing the activity of digestive enzymes in the intestine, thereby enhancing the digestibility of feed (38). After that, it has been proposed that we want to improve the utilization rate of Yu-Ping-Feng polysaccharides, so they used ultra-fine pulverization technology to ultrafine pulverize Yu-Ping-Feng polysaccharides and added the ultra-fine pulverized polysaccharide to broiler feed at the same dosage, which was found to be twice the efficacy of ordinary Yu-Ping-Feng polysaccharides, which also could improve the average body weight, survival rate, and reduce the feed to meat ratio of broilers (*p < 0.05*) (39). Other experiments found that after the addition of Yu Ping Feng polysaccharide to the diet, the body weight of ducks in the experimental group increased by 130.44 g/each compared to the control group, proving that Yu-Ping-Feng polysaccharides also played a role in promoting the growth and development of waterfowl (40). These results suggest that Yu-Ping-Feng polysaccharides has a certain promotion effect on the production performance of chicks, broilers, and waterfowl.

### 3.3. Effect of Yu-Ping-Feng polysaccharides on the production performance of other animals

In earlier times, local veterinarians commonly used Yu-Ping-Feng polysaccharides to treat various conditions in domestic animals, such as excessive sweating, night sweats, nocturnal sweats, and constipation (41–45). In modern animal production, Yu-Ping-Feng polysaccharides and its related extracts are commonly added to livestock and poultry diets as feed additives.

Some studies have shown that the addition of Yu-Ping-Feng polysaccharides to the diet can significantly improve the slaughter rate of fattening cull cattle and have some fattening effect (46, 47). It can also change the activity of digestive enzymes in the digestive tube and improve feed utilization. Some studies proved that the activity of digestive enzymes such as rumen cellulase, wrinkled gastric protease, and small intestine pancreatic protease was significantly increased in castrated cows after the addition of Yu-Ping-Feng polysaccharides to the diet of fattened castrated cows and that the enzyme activity gradually decreased with the postponement of the digestive tube; moreover, the daily weight gain, net weight, percentage of high-grade meat and net meat rate of fattened cows were all improved (48, 49). Other studies have shown that the appropriate amount of Yu-Ping-Feng polysaccharides can strengthen the absorption of feed nutrients in the digestive system of dairy cows and improve the milk production capacity of dairy cows (50, 51). In conclusion, Yu-Ping-Feng polysaccharides has been shown to enhance the productive performance of bovine.

In one experiment, goats in three groups were fed a normal diet, Yu-Ping-Feng medicinal residue + diet, and Yu-Ping-Feng medicinal residue fermented matter + diet; the results showed optimal improvement in meat quality and mutton odor taste in the goats fed the Yu-Ping-Feng ferment compared to the control group (52). Another study found that fermented Yu-Ping-Feng polysaccharides could promote the formation of beneficial digestive tract flora, increasing the content of fatty acids in muscle, and promoting growth in black goats (53). It was confirmed that the rumen-fermented Yu-Ping-Feng polysaccharides has more nutritional effects. It can be seen that both Yu-Ping-Feng polysaccharides and its fermentation products can improve the production performance of goats to different degrees.

**Figure 2:**
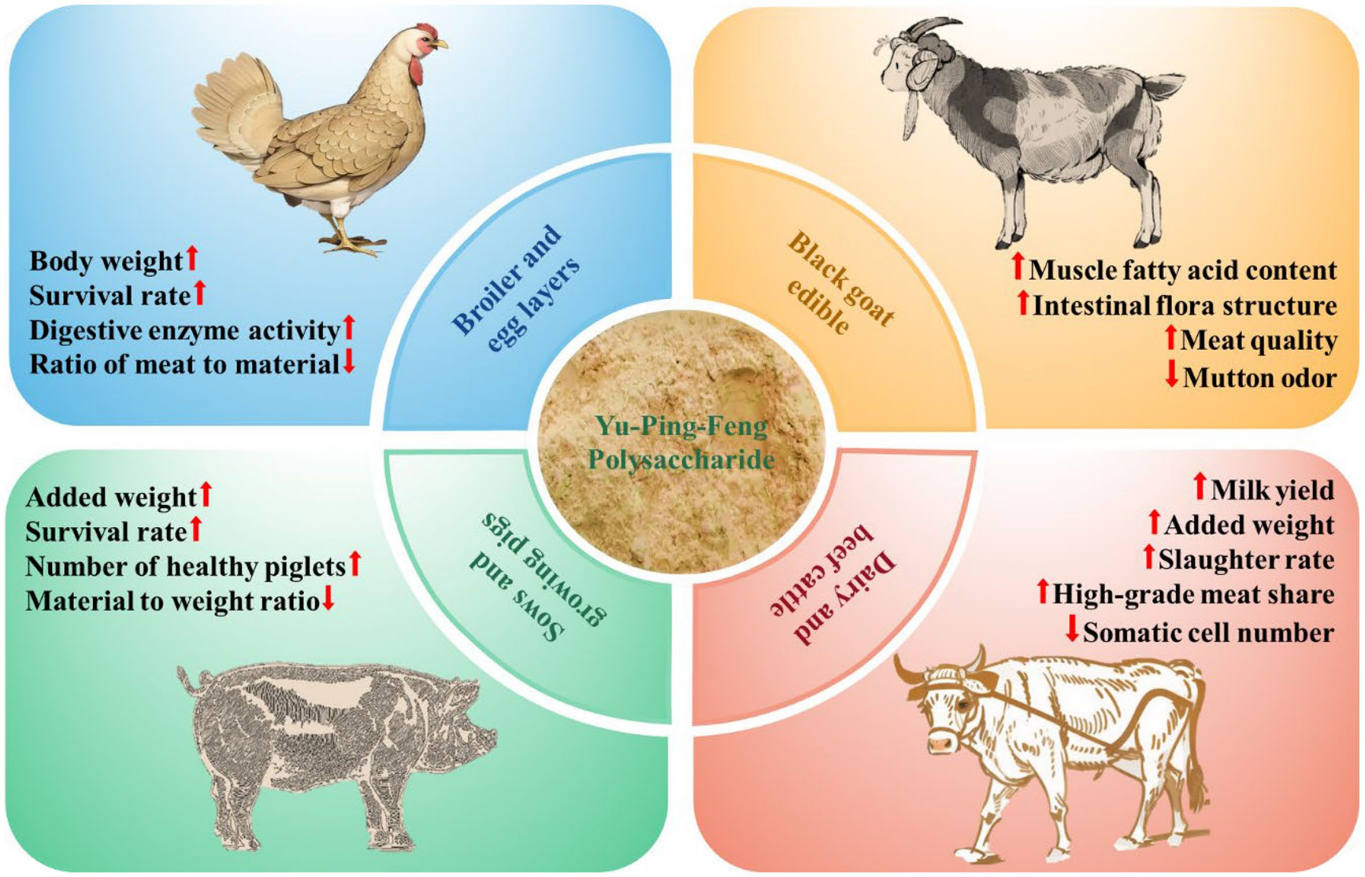
The impact of Yu-Ping-Feng polysaccharides on the production performance of economically-relevant animals. Upward arrows indicate an increase, while downward arrows indicate a decrease.

As evident from the above, Yu-Ping-Feng polysaccharides can enhance the production performance of various economically important animals (Figure 2). Serving as a green feed additive, Yu-Ping-Feng polysaccharides hold significant potential for development and offer broad prospects.

## 4. The role of Yu-Ping-Feng polysaccharides in animal immunity

The strength of the animal’s immune directly affects the animal’s health. Yu-Ping-Feng polysaccharides, a traditional Chinese medicine compound, can have a protective effect on the immune system of the animal body (54).

### 4.1. Effect of Yu-Ping-Feng polysaccharides on the immune performance of poultry

Chicks can gradually develop their immune barrier from the time they hatch out of their shells. In this process, the immune performance of chicks can be enhanced by feeding some antibiotic-free probiotics or natural herbs, thus improving the production efficiency at a later stage. It has been shown that the addition of Yu-Ping-Feng polysaccharides to chick diets could strengthen the antioxidant enzyme activity in chicks, effectively eliminate excess free radicals in the body and improve their immune capacity (55–57). Subsequently, have study found that the moderate addition of Yu-Ping-Feng polysaccharides to the diet of chicks could improve their spleen lymphocyte ratio (58). In addition, feeding chicks with a multipotent compound formulation using Yu-Ping-Feng polysaccharides as the main ingredient can likewise significantly improve the indices and immune performance of various immune organs in chicks (30, 59, 60). Another study used the “reverse evaporation method” to prepare Yu-Ping-Feng polysaccharides liposomes (YPF-PL) to study the immune characteristics of Ephedra chickens (chicks) and found that it had a significant growth-promoting effect on the spleen, small intestine, and lymphoid tissue of Ephedra chickens (chicks), with an increase in the number of immune factors and T and B lymphocyte subpopulations in peripheral blood (22, 61–65). Moreover, studies have shown that adding a combination of Yu-Ping-Feng polysaccharides, Licorice, or Yu-Ping-Feng polysaccharides and compound acidifier to White feather broiler (chicks) feed can rapidly activate the body’s immune signal and enhance disease resistance. The Yu-Ping-Feng polysaccharides may enhance the immune performance of White feather broiler (chicks) chickens by regulating immune inflammation-related signaling pathways (37, 38, 66).

Yu-Ping-Feng polysaccharides not only can enhance the immunity of poultry but also can play a synergistic immune effect of anti-stress. Related studies have shown that the polysaccharide of Yu-Ping-Feng could enhance the potency of HI antibody in animal serum, increase the ratio of CD4^+^ and CD8^+^ in cells, and enhance the immune function of animals (6). In addition, Yu-Ping-Feng polysaccharides can enhance the immune function of a chick’s thymus, bursa, spleen, and other organs, improve the immune effect of the Newcastle disease vaccine and chicken infectious bursal disease vaccine on chicks, make chicks form antibodies rapidly, and prolong the duration of antibodies (32, 67, 68). Feeding Yu-Ping-Feng polysaccharides during immunization with inactivated avian influenza oil emulsion vaccine increase antibody potency (*p < 0.05*) and increases total serum protein content (*p < 0.05*) (34). Another study found that the drug-containing of Yu-Ping-Feng polysaccharides not only effectively prevented the adsorption and invasion of *Newcastle disease virus* (NDV) on chicken embryonic fibroblasts, but also effectively reduced the virulence of NVD (69). Yu-Ping-Feng polysaccharides Oral Liquid also can enhance the immune effect of the chicken infectious bursal vaccine and strengthen the specific humoral immunity and non-specific cellular immunity of chicks (70, 71). Similarly, “Gan Lian Yu-Ping-Feng polysaccharides” which is mainly composed of Yu-Ping-Feng polysaccharides, can also effectively enhance the immunization effect of Infectious Tracheitis Vaccine (ILTV) to chicks (72).

The polysaccharide components in Yu-Ping-Feng polysaccharides are the material basis for enhancing animal immunity; it has been found during waterfowl research that Yu-Ping-Feng polysaccharides can enhance the immune function of meat ducks by increasing the content of immune factors and improving thymic function (40). In addition, after egg quail were vaccinated against avian influenza, a decoction of 0.5% Yu-Ping-Feng polysaccharides was added to water and fed for 10 days to increase the antibody potency in the serum and maintain keep it at a high antibody level (73).

### 4.2. Effect of Yu-Ping-Feng polysaccharides on the immune performance of pigs

During the growth of piglets, the addition of 0.8% of Yu-Ping-Feng polysaccharides to the diet resulted in significantly higher levels of serum lymphocyte positivity, complement (C3, C4), immunoglobulins (IgA, IgM, IgG), interleukins (IL-2) and γ-interferon (IFN-γ), and T-lymphocyte subsets (CD3, CD4, CD8) compared to the blank group (*p < 0.05*) (26, 27). When 400 mg/kg of Yu-Ping-Feng polysaccharides was added to the diet of piglets, an enhanced immune response of animals to swine fever virus was observed. At the same moment, when added at 600 mg/kg, the immunoglobulin content in the serum of piglets increased (74). Moreover, Gong et al. found that both 0.15% probiotics and 0.04% Yu-Ping-Feng polysaccharide could enhance the immunity of piglets, but the combination of the two was more effective (29).

According to the relevant literature, the effects of the addition of the traditional Chinese medicine compound Yu-Ping-Feng polysaccharides on the immune performance of different species of economic animals are summarized as follows. As shown in (Table 1), Compound Yuping Feng polysaccharides made from different herb dosage ratios can affect animals of this species to varying degrees, depending on the dosage as well as the duration of the dosage. “+” represents positive impacts and “-” represents negative impacts.

**Table 1:** The role of Yu-Ping-Feng polysaccharides in the immune performance of animals.

Animals	Composition	Optimal Dose	Time	Immunological parameters	Reference
Newcastle disease LaSota vaccine drinking water immunization (double dose) (chicks)	YPF(3:1:1)-P+ Party Ginseng (2)	2 mL/ kg	7 d	+ (Thymus, spleen, bursa index, SH-Px, SOD, RBC-CR1R), - (MDA)	(55–57)
Pathogen-free broiler (chicks)	YPF(3:1:1)-P+ Angelica (1)+Epimedium (1)	1.00%	7 d	+ (Thymus, spleen, bursa index, IL-2, IFN-γ, Ig G, Ig M)	(59)
Pathogen-free chicks (spleen)	YPF(3:1:1)-P	500 mg/ ml	48 h	+ (Splenic T and B lymphocytes)	(58)
Ephedra chickens (chicks)	YPF(3:1:1)-PL (Yu-Ping-Feng polysaccharides liposomes)	200 ~ 400 mg/kg	2 times/ d × 7 d	+ (Avian influenza virus antibody levels, Splenic lymphocyte activity and number, Number of intestinal IELs and LPLs, Area of lymphoid follicles in the tonsils of the appendix, GM-CSF, G-CSF and M-CSF levels in peripheral blood, Proportion of CD4^+^ and CD8^+^ T lymphocytes, Bu-1 + B lymphocyte ratio)	(22, 61, 62)
White feather broiler (chicks)	YPF(3:1:1)-P	600 mg/kg	42 d	+ (Pancreas, bursa phalloides, H5 antibody, NVD antibody, Spleen + liver IFN-γ) -(Splenic TNF-α, TRAF2, Hepatic TNF-α, IκBα)	(66)
White feather broiler (chicks)	YPF(4:3:3)-P+ Licorice extract	500 mg/kg +200 mg/kg	21 d/42 d	+ (Serum H5-11, H9, H7, NDV antibody levels, Liver and spleen organ indices)	(37)
White feather broiler (chicks)	YPF(3:1:1)-P+ Compound acidifier	600 mg/kg +2000 mg/kg	42 d	+ (Liver and spleen organ indices, IL-10) - (IL-1, IL-2)	(38)
Hy-Line chickens	YPF(3:1:1)-PS	5.6 mL	1 times/ d × 3 d	+ (Spleen, thymus and phalanx organ indices, Serum HI antibody, Peripheral lymphocyte proliferation and distribution, Percentage of CD4 and CD8 T lymphocytes)	(6)
Hyland White (chicks)	YPF(2:2:1)-P	4.5 g/ d	14 d	+ (Spleen, bursa phalloides, thymus, HI antibody, Peripheral blood T and B lymphocytes)	(32)
Isha brown eggs (chicks)	YPF(3:1:1)-P(Oral Liquid)	1 mL/ kg	3 weeks	+ (Chicken ND, IBD vaccine antibody levels, Spleen, bursa and thymus organ indices)	(67)
Isha brown eggs (chicks)	YPF(3:1:1)-P(Oral Liquid)	4 mL	3 weeks	+ (Effectiveness of IBD vaccine immunization)	(70)
1 day old laying hens (chicks)	YPF(3:1:1)-P	2 mL/ L	42 d	+ (Newcastle disease antibody potency, Spleen, bursa index)	(68)
Cobalt Fast Large White Feather Broiler (chicks)	YPF(3:1:1)-P	1%	44 d	+ (Avian influenza HI antibody potency, Total serum protein content)	(34)
Jingbai Chicken (chicks)	YPF(3:1:1)-P	1%	12 d	+ (Serum IBDV antibody levels, Number of CD4^+^, CD4^+^/CD8^+^ values, Peripheral blood macrophage activity)	(71)
Jingbai Chicken (chicks)	YPF(3:1:1)-P+ Heart-throbbing lotus (2)+Licorice (1)	2 mL/ L	6 d	+ (IFN-γ content, ILT antibody titer, Immune organ indicators) -(IL-4 content)	(60)
Cherry Valley meat duck (duckling)	YPF(3:1:1)-P+ Jujube + Angelica	2 mL/ L	3 weeks	+(Avian influenza (H5N1, Re-5) antibody level, Thymic organ index, IL-2, IFN-γ, IgG, IgA immune factor levels)	(40)
Egg quail	YPF(3:1:1)-P	0.50%	90 d	+ (Inactivated avian influenza virus vaccine (H5N1 subtype, Re-1 strain) antibody levels)	(73)
Du Changda ternary crossbred pigs (weaning piglets)	YPF(3:1:1)-P	0.80%	52 d	+ (Serum IgG, IgM, IgA, C3 and C4 levels, IL-2, IFN-γ, Lymphocyte positivity rate, CD4+/CD8 + values)	(26)
Weaned piglets	YPF(3:1:1)-P+ Acanthopanax + *Lycium barbarum* leaves	0.80%	42 d	+ (Serum IgG, IgM, IgA levels)	(27)
Du Changda ternary crossbred pigs (weaning piglets)	YPF(2:2:1)-P	600 mg/ kg	21 d	+ (Swine fever antibody level, Serum IgG, IgM, IgA levels)	(74)
Dugong Ternary crossbred growing pigs	YPF(3:1:1)-P+ Probiotics	0.04% + 0.15%	60 d	+ (Serum IgG, IgA levels)	(29)

Based on the available evidence, it appears that Yu-Ping-Feng polysaccharides can be utilized to improve the immune function in poultry and pigs. Various studies have demonstrated that Yu-Ping-Feng polysaccharides has multiple immune-enhancing effects, including the stimulation of immune cell proliferation and cell cycle distribution, increasing protein content in serum, and maintaining the potency of immune vaccines.

## 5. Outlook

Yu-Ping-Feng polysaccharides has promising market potential in animal production. Extensive experimentation has demonstrated the value of Yu-Ping-Feng polysaccharides, whether used simply as a feed additive or in combination with other substances, in promoting animal growth and development, as well as enhancing disease immunity. In the context of modern animal production processes, the use of Yu-Ping-Feng polysaccharides or its derivatives can yield significant productivity gains, thereby ensuring improved profitability for farms.

The polysaccharides from Yu-Ping-Feng have positive effects in the field of economic animal breeding, but there are still some issues: (1) In poultry and livestock farming, Yu-Ping-Feng polysaccharides have great potential as a natural feed additive for antibiotic substitution. However, there are limited comparative experiments between antibiotics and Yu-Ping-Feng polysaccharides on animals, and there is a lack of scientific data to support this. More research is needed to confirm the substitutive ability of Yu-Ping-Feng polysaccharides for antibiotics. (2) Yu-Ping-Feng polysaccharides can be extracted from the medicinal residue byproducts of Yu-Ping-Feng prescriptions produced by traditional Chinese medicine companies. However, the utilization of these medicinal residues by traditional Chinese medicine companies is not sufficient. It is hoped that secondary development and utilization of these residues can turn waste into valuable resources. Resolving the above-mentioned issues can further enhance the understanding and utilization of Yu-Ping-Feng polysaccharides, which is of significant importance for non-antibiotic animal breeding.

## Author contributions

HC: Conceptualization, Formal analysis, Methodology, Visualization, Writing – original draft, Writing – review & editing. YZ: Formal analysis, Supervision, Writing – review & editing. HY: Conceptualization, Supervision, Writing – review & editing. SC: Supervision, Writing – review & editing. ZM: Conceptualization, Methodology, Supervision, Writing – original draft, Writing – review & editing. HL: Conceptualization, Supervision, Writing – review & editing.

## Funding

The author(s) declare financial support was received for the research, authorship, and/or publication of this article. This work is supported by the National Natural Science Foundation of China (No.32002156), the Natural Science Foundation of Guangdong (No.2019A1515110454), and the Key Area Research and Development Program of Guangdong Province (2019B110209005).

## Conflict of interest

The authors declare that the research was conducted in the absence of any commercial or financial relationships that could be construed as a potential conflict of interest.

## Publisher’s note

All claims expressed in this article are solely those of the authors and do not necessarily represent those of their affiliated organizations, or those of the publisher, the editors and the reviewers. Any product that may be evaluated in this article, or claim that may be made by its manufacturer, is not guaranteed or endorsed by the publisher.
